# How is sedation provided for percutaneous dilatational tracheostomy in English ICUs?

**DOI:** 10.1186/cc9769

**Published:** 2011-03-11

**Authors:** P Hampshire, L McCrossan

**Affiliations:** 1 Royal Liverpool and Broadgreen University Hospital, Liverpool, UK

## Introduction

Percutaneous dilatational tracheostomy (PDT) is commonly performed at the bedside in the ICU. Patients in the ICU often have multiple organ dysfunction, causing alterations in drug effects and metabolism. Alterations in sedative drug handling may make them vulnerable to awareness during PDT. Up to 40% of patients in the ICU report some awareness whilst receiving neuromuscular receptor blocking drugs [1] - these drugs are usually employed when performing PDT. Depth of anaesthesia monitoring may prevent awareness and has been used during PDT [2]. Various depths of anaesthesia monitors are available, including the bispectral index monitor (BIS), the Narcotrend Index and the State and Response Entropy, derived from the EEG. We report the results of a telephone survey on the sedation given for PDT in English ICUs.

## Methods

We contacted 240 adult ICUs in England by telephone. Two hundred and twenty-four units (93%) participated.

## Results

Two hundred and fourteen units (95%) perform PDT as their first-choice technique. Units that do not practice PDT (*n *= 10, 5%) perform open surgical tracheostomy. Most ICUs use simple infusions of propofol via standard infusion pumps during PDT (*n *= 202, 94%), and give additional boluses of propofol if necessary. In seven units (3.3%) anaesthesia is provided using intermittent boluses of propofol, without a background infusion. This may be of concern given that one study reported awareness during rigid bronchoscopies [3] and all the patients who reported awareness were anaesthetized using intermittent boluses of propofol. Nine units (4.2%) reported using a BIS during PDT. Three ICUs have used a BIS on a trial basis, but have discontinued. One reason given for discontinuing using a BIS was that it 'made no difference to the amount of sedation' during PDT.

## Conclusions

Depth of anaesthesia monitoring is not widely used in English ICUs during PDT. It is unclear whether a BIS is effective for monitoring depth of anaesthesia during PDT, and further studies are needed to clarify this.
